# A phenotypic screen identifies xanthohumol and other flavonoids as killers of bladder cancer

**DOI:** 10.1016/j.prenap.2025.100236

**Published:** 2025-04-25

**Authors:** Michael J. Bolt, Jessica Oceguera, Alejandra Rivera Tostado, Christopher D. Candler, Elina Mosa, Kazem Safari, Maureen G. Mancini, Michael A. Mancini

**Affiliations:** a Department of Molecular and Cellular Biology, Baylor College of Medicine, Houston, TX, United States; b GCC Center for Advanced Microscopy and Image Informatics, Houston, Houston, TX, United States; c Center for Translational Cancer Research, Institute of Biosciences & Technology, Texas A&M University, Houston, TX, United States

**Keywords:** Cell Paint, Phenotypic screening, Bladder cancer, Xanthohumol

## Abstract

Bladder cancer accounts for 4 % of cancer diagnoses in the US. Current treatments primarily involve trans-urethral resection of bladder tumors (TURBT) and immunotherapy with Bacille Calmette-Guerin (BCG). Despite the efficacy of TURBT, issues with residual tumors persist. We chose to utilize Cell Painting to screen a set of 244 flavonoid compounds for bladder cancer cell toxicity. Using multiparametric high content analysis termed SPACe, we discover promising candidates underscoring the potential of flavonoids in targeting bladder cancer cells and elucidating their mechanisms of action. Notably, compounds such as xanthohumol show promise in reducing cancer cell viability by altering lipid metabolism. We further show the effectiveness of hit compounds in subsequent spheroid and chorioallantoic membrane systems. Overall, this research emphasizes the role of innovative screening methods in drug discovery and potential synergistic effects of combining flavonoids with existing therapies like BCG for improved bladder cancer treatment outcomes.

## Introduction

Bladder cancer (BC) is the 5th most common cancer in the United States, with a 3:1 preference for males. The American Cancer Society estimates over 75,000 new cases with over 16,000 deaths per year. BC is most often a result of chronic toxin exposure, such as cigarette smoke, fungicides, plastics, and aromatic amines from highly processed foods. In 95 % of cases, BC transitions transitional epithelium into low grade tumors that have not invaded the nearby muscle. These tumors are termed non-muscle invasive bladder cancer (NMIBC). Currently, most treatments for NMIBC are structured around surgical removal of the cancerous mass combined with perioperative therapy. NMIBC prefers usage of trans-urethral resection of bladder tumor (TURBT) to radical cystectomy (RC), especially for low and intermediate risk categories [1]. TURBT is usually paired with perioperative chemotherapy and maintenance therapy with intravesical Bacille Calmette-Guerin (BCG), a vaccine made from a weakened tuberculosis-related bacteria which activates the immune system at the bladder epithelium, once the tumor is surgically removed. In high risk categories, BCG is recommended as the primary treatment with the potential to continue use as a maintenance therapy [2].

The usage of TURBT is significantly important for treatment of NMIBC, but is not sufficient, due to its relative imprecision when fully-extracting tumors [3]. In fact, it had been found that residual tumors (36–86 %) have been identified at the original resection site [4]. How-ever, it was shown that when combined with perioperative chemotherapy, recurrence rates were reduced in low-risk patients. Survival rate for NMIBC is depended upon tumor type and grade, with T1HG (high-grade tumors that invade the submucosa) having the highest cancer-specific mortality (CSM) at 19.52 % and TaLG (low-grade tumors that are non-invasive papillary tumors) with the lowest CSM at 3.76 % [5]. While NMBIC is the most common form of BC at 70 % of new cases, it can progress to muscle-invasive bladder cancer (MIBC) and further to metastatic bladder cancer, both comprising 25 % and 5 % of newly diagnosed cases, respectively. MIBC itself is particularly more dangerous than NIMBC, with a high rate of recurrence and a 5-year mortality rate of about 50–70 % [6] even in combination with new immunotherapy approaches.

Screening for therapeutic compounds against BC is not a novel concept. A screen looking for differential effects based on BC subtype was focused on drug repurposing libraries [7]. This screen utilized CellTox green, a simple death assay, to discover their toxic hits across 36 BC cell lines. While their findings were consistent with in vivo studies and clinical trials on pathways important for certain subtypes of BC, the hits discovered in this screen have yet to materialize as therapeutic options. Another survey against a broad spectrum of 23 BC cell lines was also used in a repurposed drug screen for potential use in therapeutic intervention. In this case, while a hit compound, clofarabine, has been tested in childhood leukemia treatments and inhibited BC growth across subtypes in both cell lines and PDX models, this assay was performed without replicate wells for each compound. Whereas both single endpoint screens utilized libraries with compounds of known mechanisms of action, single endpoint assays can potentially result in missed opportunities.

Flavonoids are phenolic compounds commonly found in plants and have varied biological effects, including some with estrogenic activity (*i. e.*, genistein). As such, we recently screened a set of 244 flavonoid compounds by multiparametric high content analysis for their estrogenic activity [8]. Data from this flavonoid screen identified multiple compounds that exhibited a possible toxic effect of flavonoid compounds in both cell culture and zebrafish assays [8]. Indeed, several flavonoids have been shown to have any anti-tumor effects in BC (tectoridin [8] and hesperetin [9]), and many other cancer types (genistein in breast [10], baicalin in lung [11], diosmetin in colon [12]). Therefore, we set out to specifically test our flavonoid library for a potential anti-tumorigenic effect in a bladder cancer context chiefly because many flavonoids are consumed through daily vegetable intake, resulting in high concentrations in urine [13] that may potentially effect the bladder tumor microenvironment. Using the Cell Painting approach, we screened the same flavonoid library in several BC cell lines (5637, UMUC3, RT4) that represent different subtypes (basal, neuroendocrine, and luminal, respectively) to not only identify compounds that kill bladder cancer cells, but also to obtain insights into their mechanism(s) of action (MOA). Here, we report that within the library we identified a set of 6 flavonoids that killed bladder cancer cell lines, including two known toxic flavonoids (flavopiridol and rotenone). Furthermore, additional study revealed hits could be subdivided into those causing either DNA damage or altered mitochondrial/energy function. Importantly, we also identified 3 flavonoids in a more physiological cell culture assay using BC spheroids that specifically resulted in decreased growth, but with no effect upon primary bladder fibroblast spheroids. Lastly, in an orthogonal avian egg assay, xanthohumol was specifically shown to reduce tumor growth in a chorioallantoic membrane model. Taken together, these results further highlight the benefits of phenotypic screening by Cell Painting [14] along with follow-on assays to identify functional hits that may have a therapeutic potential.

## Materials and Methods

### Cell lines

5637, UMUC3, J82, and RT112 cells were grown and treated in Roswell Park Memorial Institute 1640 (RPMI) media supplemented with 10 % FBS. RT4 cells were grown and treated in McCoy’s 5A (Modified) media supplemented with 10 % FBS. The primary bladder fibroblasts were grown in Fibroblast Growth Media from ATCC containing FGF (5 ng/ml), ascorbic acid (50ug/ml), hydrocortisone (1ug/ml), insulin (5ug/ml), L-glutamine (7.5 mM) and 2 % FBS. 5637, UMUC3, J82, RT4, primary bladder fibroblast cell lines were purchased through the American Type Culture Collection (ATCC.org). The RT112 were obtained from Zhi Tan at Baylor College of Medicine.

### Flavonoid library and treatment

Flavonoid library (Targetmol #L7700) was added to 384-well plates (PerkinElmer) containing cells (3000 cells/well) using an Echo 650 Acoustic Liquid Handler (Beckman Coulter) to a final concentration of 10uM. Plates were then incubated for 72 h. Two replicate plates with 2 wells per plate per compound were used for the screen. For all assays, DMSO is used as the vehicle control.

Hit compounds were individually purchased from Targetmol for follow-up studies.

### Cell painting assay and analysis

Treated plates had their media removed and media containing Mitotracker^™^ (ThermoFisher) were added to the wells for 30 minutes. Cells were fixed (4 % EM grade formaldehyde in PBS) for 20 minutes followed by three 5-minute PBS washes. Cells were incubated with staining mixture (1ug/ml DAPI (ThermoFisher), Syto14 (ThermoFisher), Phalloidin 568 (ThermoFisher), 0.1 % Triton TX-100 (ThermoFisher) in PBS) for 30 minutes followed by three 5-minute PBS washes. The wells are then left in 40ul PBS. Plates were imaged on the ImageXpress Micro (Molecular Devices). Images were analyzed using our SPACe, our custom Cell Painting platform [14].

### yH2AX Immunolabeling

Cells were seeded into a 384-well optical bottom microtiter plate. Following compound treatment for 24 h, cells were fixed in 4 % EM grade paraformaldehyde (Electron Microscopy Sciences) in Ca++/Mg++ PBS for 20 minutes and permeabilized for 15 min with 0.5 % Triton X-100. Cells were incubated at room temperature with 5 % milk (ThermoFisher) in PBS at room temperature for 1 h, followed by incubation with yH2AX antibody (Cell Signaling Technologies) overnight at 4°C. Cells were washed and incubated with Goat anti-rabbit Alexa Fluor 647 (ThermoFisher) for 1 h at room temperature, followed by post-fixing in 4 % EM grade formaldehyde in PBS for 10 min, and finally by counterstaining with DAPI (1ug/ml). The plate was then imaged on Yokogawa CV8000. Each test case was performed on replicate plates containing quadruplicate wells of a given compound and dose.

### Nanolive experiments and quantitation

As performed in Perez-Monteroa et al [15]. 5637 and UMUC3 cells were plated on IBIDI 35 mm dishes and allowed to grow to 70–80 % confluence. Media was then changed and either DMSO or flavonoid compounds were added at 10uM concentration. A DIC lid (Nanolive CX-DICL10) was added, and the dishes were put on the instrument. A 5 × 5 field grid was imaged from each dish every 10 minutes for 20 hours on the Nanolive 3D Cell Explorer. Nanolive assays were performed in duplicate. Quantitation of lipid dry mass was performed in the Nanolive Eve analytics platform. Lipid count was performed on a custom CellProfiler pipeline that segments nuclei based on DAPI signal and lipid droplets based on Bodipy (ThermoFisher) staining. Results are the aggregate of two individual experiments. Each test case was done on replicate plates with each plate containing quadruplicate wells of a given compound and dose.

### Flavonoid growth assay

Cells were plated (3000 cells per well) in a 384-well plate. After 24 hr, cells were treated with flavonoid compound for 72 hours. Cells were then fixed and DAPI labelled (as above) and images on the ImageXpress Micro using a 4X lens to image the entire well. Nuclear count was established using a custom CellProfiler pipeline (available upon request). Each test case was done on replicate plates with each plate containing quadruplicate wells of a given compound and dose.

### Spheroid growth assay and analysis

Spheroid generation was performed as previously published [16]. Succinctly, 1 % agarose was melted down then added to a 96-well plate which was then briefly swirled to create a 3D ‘cup’ for cells to collect in the center and grow into a spheroid. 3000 cells per well of a given cell line were added to the plates. 24 h after cells were added, flavonoid compound was added. Spheroids were then imaged every 48hrs for 6 days on the Yokogawa CV8000. For analysis, the best focal plane (largest in focus area) of each spheroid was chosen, and the area of the sphere was measured using a custom pipeline using CellProfiler. Each test case was done on replicate plates with each plate containing duplicate wells of a given compound and dose.

### Generation of EGFP-Luc positive cells

pHAGE-EF1a-EGFP-Ires-Luc-Blast vector was constructed in the ACE3M core at Baylor College of Medicine. Lentivirus was packaged from this vector by co-transfecting 293T cells with psPAX2 (Addgene #12260) and pMD2.G (Addgene #12259), viral supernatant was collected 48 h after transfection and filtered through a 0.45um syringe filter. RT4 or 5637 cells were seeded at 30 % confluency in a 6-well plate the day before infection, cells were incubated overnight with 1 ml viral supernatant and 4ug/ml polybrene. The cells were then selected with 10ug/ml blasticidin for 5 days. 50 % of the cells were EGFP positive after blasticidin selection. EGFP positive cells were further enriched to more than 95 % by flow cytometry sorting.

### CAM preparation and tumor seeding

All chicken egg CAM procedures, including humane euthanasia of chicken eggs, were conducted in accordance with the 2020 guidelines of the American Veterinary Medical Association (AVMA) and with the approval of Baylor College of Medicine’s institutional animal care and use committee (Protocol AN-7103). Specific Pathogen-Free (SPF) fertilized chicken eggs were placed in a humidity-controlled rocking incubator at 37 °C on the day of arrival, for a period of seven days. All subsequent egg work was performed inside of a laminar flow hood. The chicken egg CAM was prepared according to our established protocols [17,18]. Briefly, a window opening was created on the eggshell and the inner shell membrane was removed using fine-point curved forceps to expose the CAM. A silicone ring was placed on top of the CAM for each egg and the opening was taped shut using clear tape. The eggs were placed in a Styrofoam incubator for 1–2 h while the cells for engraftment were prepared. EGFP-luciferase expressing RT4 and 5637 bladder cancer cells were harvested from tissue culture flasks and re-suspended at a concentration of 5 × 10^5^ cells per 60 μl in PBS supplemented with magnesium and calcium. Matrigel basement membrane matrix (Corning, Corning, NY) was added to the cell suspensions to bring the total volume to 100 μl per egg engraftment. The eggs were transferred to the laminar flow hood and the tape was peeled back to add a 100 μl cell suspension applied topically to the center of the silicone ring area on top of the CAM. The eggshell opening was re-taped and eggs were returned to the humidified, temperature-controlled Styrofoam incubators for the remainder of the experiment. Embryo viability was examined daily, and any expired eggs were removed and disposed promptly.

### In vivo imaging, tumor treatment and harvest

On day three post engraftment, eggs were removed from the incubators and received 50 μl of 15 mg/ml D-Luciferin (Goldbio, St. Louis, MO) directly over the cell engraftments. *In situ* luminescence of the tumors was measured using an IVIS Lumina III in vivo imaging system (Revvity, Waltham, MA). Longitudinal imaging was performed on all tumors growing within the CAM and were subsequently randomized into different treatment groups. Treatment with vehicle control or flavonoids was carried out immediately following tumor randomization using a volume of 50 μl per egg. The chick eggs were returned to the incubators and tumors were allowed to grow for an additional four days with treatment and luminescence imaging on days three, five, and seven. Following imaging on day seven, tumors were excised from the CAM and fixed in 10 % neutral-buffered formalin at 4 °C. After 48 hours, the formalin was replaced with 70 % ethanol and fixed tissues were submitted to the Human Tissue and Acquisition Pathology core at Baylor College of Medicine for tissue processing, embedding, sectioning, and staining with hematoxylin and eosin.

### Graphics and statistical analysis

**Fig. 1.**
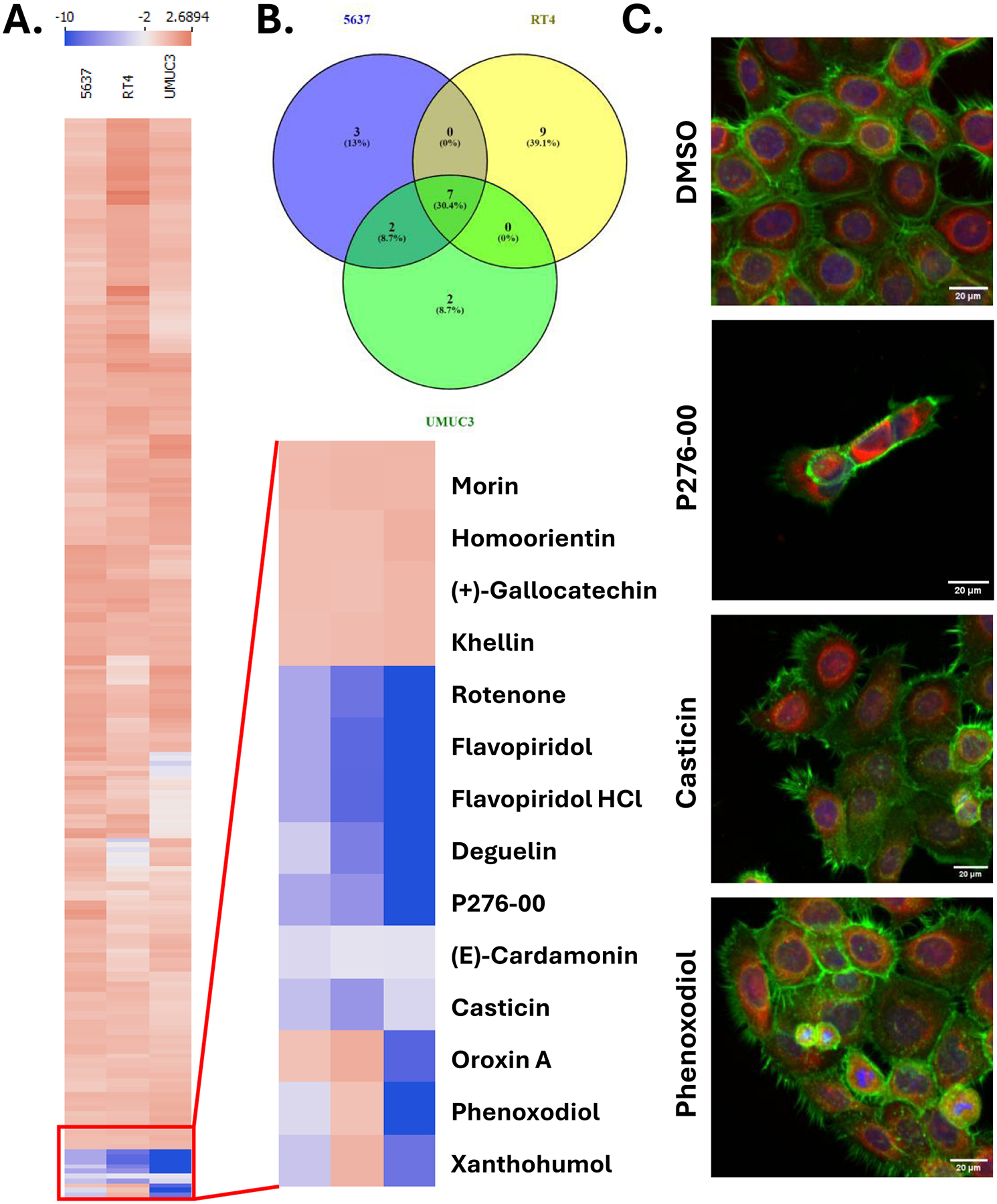
A screen to discover cytotoxic flavonoids against bladder cancer. Heatmap of the Z-score normalized nuclear count for flavonoid compounds in 5637, RT4, and UMUC3 cell lines. The inset is an enlargement of the hits portion of the heatmap. B) A Venn diagram of hits from all 3 cell lines. C) Example images of cell painting of 5637 cells treated with DMSO, P276–00, casticin, or phenoxodiol.

**Fig. 2.**
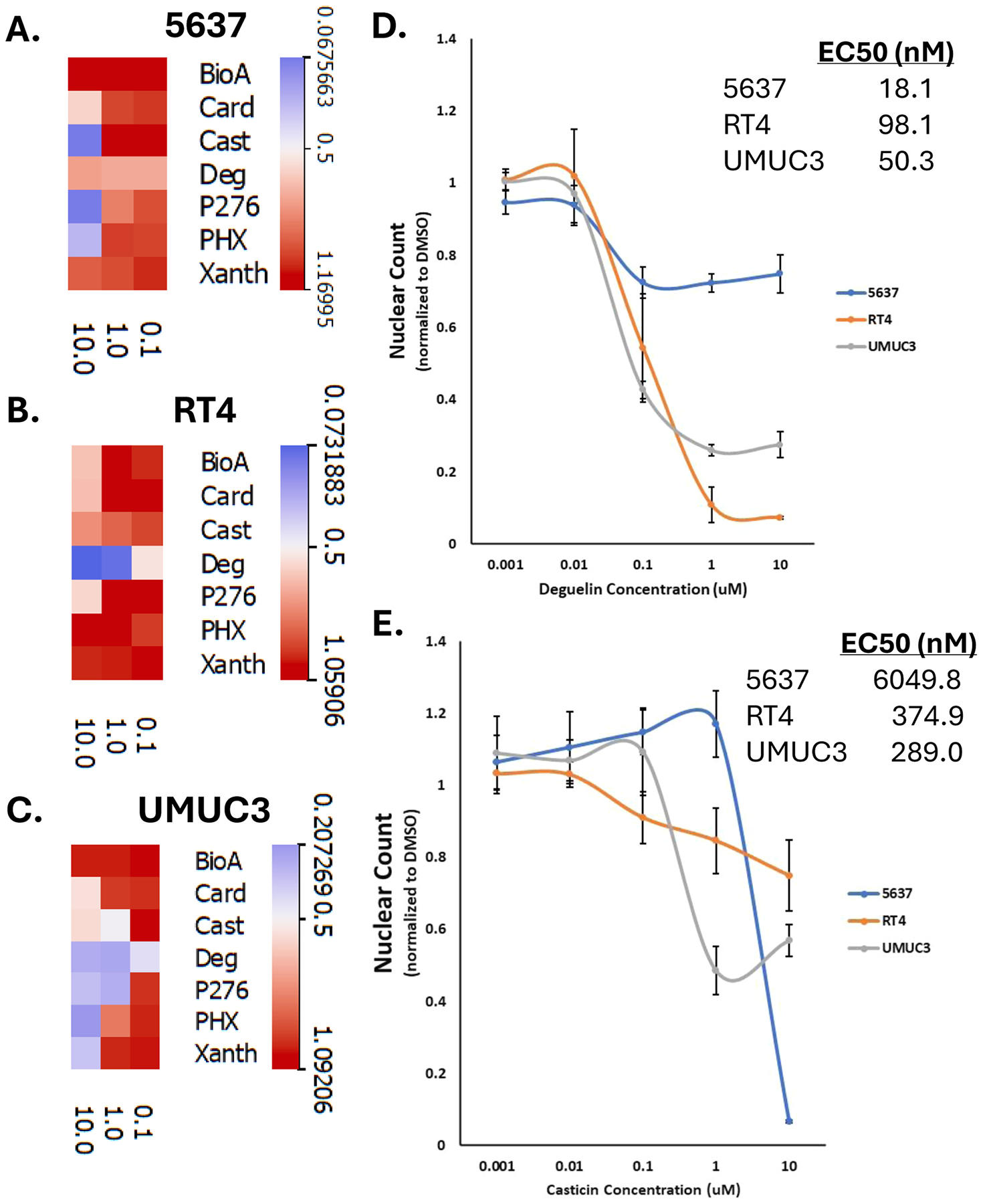
Flavonoids kills bladder cancer cells in a dose dependent manner. Heat maps of nuclear count for our toxic compounds in **A)** 5637, **B)** RT4, and **C)** UMUC3 bladder cancer cells. The data is normalized to the DMSO control. **D)** Dose response curves and EC50 values for deguelin’s effect on nuclear count across the three bladder cell lines. **E)** Dose response curves and EC50 values for casticin’s effect on nuclear count across the three bladder cell lines.

All graphics were made using Microsoft Excel unless otherwise stated. The heat maps in Figs. 1, 2, and 4 were made in the data analysis software Orange. p-value was determined using student’s *t*-test for single comparisons or one-way ANOVA for independent samples and a Tukey HSD post-hoc test for comparisons across multiple samples.

## Results

### A cell paint-based high throughput screen to discover toxic flavonoids

We chose to utilize the increasingly popular Cell Painting^15^methodology to identify compound effects upon cancer cell biology, not only using cell counts as an end point. We treated 3 bladder cancer cell lines (5637, RT4, and UMUC3) with a library of 244 flavonoid compounds (TargetMol L7700) at 10uM for 72 h. We chose these three cell lines because they represent 3 distinct types of bladder cancer based on transcriptomic profiles [19], with 5637 representing a basal/squamous phenotype, RT4 representing a luminal P phenotype, and UMUC3 representing a neuroendocrine-like phenotype. Fig. 1A shows a heatmap of the flavonoid compounds across 5637, RT4, and UMUC3 based on Z-score of nuclei count. Fig. 1B shows the overlap of the hits using a 2 Z-score cut off. While there were some compounds that increased the proliferation of the cell lines, our focus was to identify compounds that were toxic/caused decreased proliferation. Seven compounds (flavopiridol, casticin, P276–00, rotenone, phenoxodiol, cardamonin, and xanthohumol) showed decreased cell count across the 3 cell lines, while 2 compounds (deguelin and oroxin A) showed toxicity in only 5637 and UMUC3 cells. Examples of cell painting images of the 5637 cells treated with DMSO, P276–00, casticin, or phenoxodiol are shown in Fig. 1E.

We utilized two control compounds within the screen, actinomycin D and berberine chloride. actinomycin D was used as a positive cell toxicity control and resulted in high levels of toxicity across cell lines (Supplemental Figure 1A). Berberine chloride is known to cause phenotypic changes in mitochondria and was used as a Cell Painting control. Supplemental Figure 1B shows the effect of berberine chloride on bladder cell mitochondria confirming that our assay is working correctly and is stable across the plates. Interestingly, the UMUC3 cell line was less susceptible to the mitochondrial changes usually induced by berberine chloride, consistent with previous results from our lab suggesting that while most cell lines are affected by berberine chloride, some cell lines are resistant [14].

We next wanted to use our CP outputs to determine how the toxic flavonoids were disrupting cell proliferation. We noticed a group pf the compounds (P276–00, phenoxodiol, and casticin) disrupted features pertaining to the DAPI signal, including changes to nuclear area, EFC ratio (an exacting measure of circularity [20]), DAPI intensity and DAPI contrast (Supplemental Figure 2A) Our results suggest that these compounds may be cytotoxic due to DNA damage or challenges to replication. Another group of compounds (deguelin, rotenone, and xanthohumol) showed more changes to the mitochondrial features, including mitochondrial area, mitochondrial intensity, and mitochondrial contrast (Supplemental Figure 2B). This suggests mitochondrial dysfunction that can lead to energy imbalance and possible disruption of ATP production. Out of the 244 flavonoid compounds, only those that significantly impacted cell number caused significant changes to phenotypic measurements.

Next, we wanted to determine the dose-dependent effect of the toxic compounds on the bladder cancer cells lines. For this purpose, we tested 5 of the 7 consensus compounds (flavopiridol and flavopiridol HCl already have been shown to be very toxic to cells [21–24], phenoxodiol and xanthohumol showed toxicity biochannin A (a compound that showed toxicity only in RT4). We treated each cell line with 3 concentrations of the flavonoids (100 nM, 1uM, 10uM) for 72 h then formaldehyde-fixed and counted the cells based upon DAPI-stained nuclear segmentation. Heat maps showing the results of the compound treatment for 5637 (Fig. 2A), RT4 (Fig. 2B), and UMUC3 (Fig. 2C) showed a mixture of dose dependent (RT4 deguelin) and stagnant (5637 deguelin) effects of the flavonoid compounds on cell number. Since casticin and deguelin were the only two compounds that were still effective at 100 nM, we performed a further dose response to include 10 nM and 1 nM. Fig. 2D shows the dose response curves on cell count for deguelin across the three cell lines, including the EC50. The EC50 for deguelin varied about an order of magnitude but were all within the range of 10–100 nM as the effective dose. Fig. 2E shows the dose response curves of the cell counts following casticin treatment across the three cell lines, including the EC50. While the EC50s for deguelin are all within an order of magnitude of each other, the toxicity changed markedly, with deguelin causing a ~25 % decrease in 5637 nuclear counts, but a ~90 % decrease in RT4. Unlike deguelin, casticin had widely different EC50s across the cell lines, with it being most potent against RT4, followed by UMUC3, and finally 5637. Next, we sought to discern the mechanism of action for the flavonoid library hits.

### Casticin, P276–00, and phenoxodiol induce DNA damage in bladder cells

**Fig. 3.**
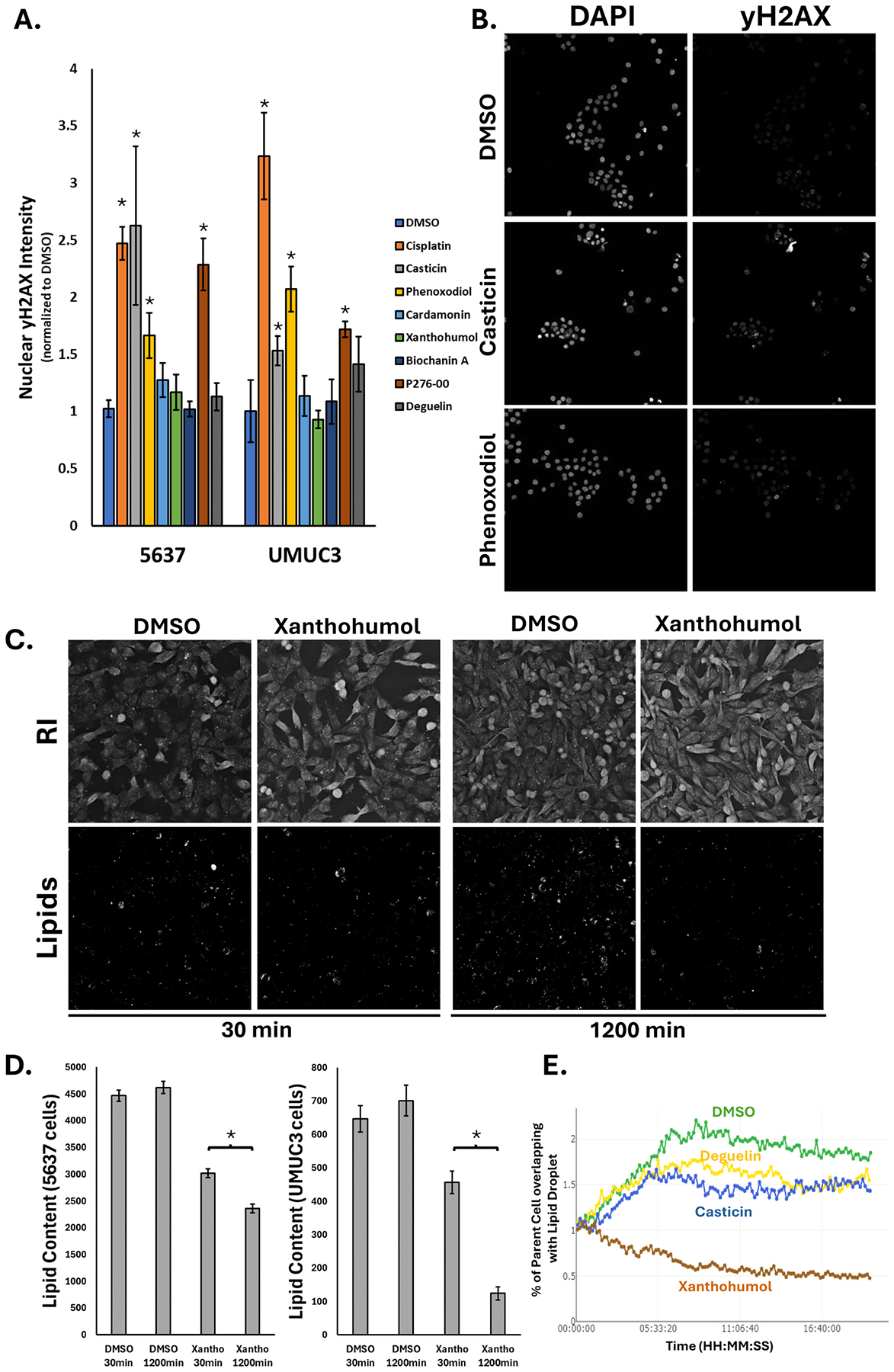
Mechanistic insights into bladder killing flavonoids. A) Bar graphs showing nuclear yH2AX intensity induced by flavonoids after 24 hr treatment in either 5637 or UMUC3 cells, normalized to their respective DMSO controls. Images are representative of staining in UMUC3. **B)** Representative images of DAPI and yH2AX labeling with flavonoid treatment in 5637 cells. **C)** Representative images of DMSO- or xanthohumol-treated refractive index maximum intensity projections from the Nanolive and thresholded images looking only at the lipids within the cells at two time points. **D)** Bar graphs quantifying the lipid content within 5637 or UMUC3 cells when treated with DMSO or xanthohumol. **E)** Line graphs quantifying the changes in % or parent cell area covered by lipid droplets over time in UMUC3 cells treated with DMSO or xanthohumol. * represents a p-value of less than 0.05.

One of the ways through which compounds can kill cells is through DNA damage that results in cessation of division followed by apoptosis. Our CP analysis (Supplemental Figure 2A) showed several of the compounds altered DAPI staining features, which led to further testing. To accomplish this, we treated 5637 and UMUC3 cells with cisplatin or hit compounds for 24 hours, then immunolabeled cells with an antibody to yH2AX, the histone mark that accumulates upon DNA damage [25]. Supplemental Figure 3A shows that our control compound, cisplatin, results in marked accumulation of nuclear yH2AX signals of both 5637 and UMUC3 cells. From our set of toxic flavonoids, P276–00, casticin, and phenoxodiol all induced yH2AX labeling (Fig. 3A) in 5637 (Fig. 3B) and UMUC3 cells. Casticin has already been shown to induce DNA damage through TOPOII inhibition [26,27], thus providing another DNA damage control for the CP assay. Deguelin, xanthohumol, and biochannin A did not induce yH2AX labeling. This data highlights how utilization of CP data can provide a base level understanding of mechanisms of action.

### Xanthohumol reduced lipid content of bladder cancer cells

To further investigate possible mechanisms of action for xanthohumol, we utilized the Nanolive refractive index live cell microscope to look for phenotypic features that change over time [28] comparing xanthohumol versus DMSO treatments. This system creates images reflecting refractive index differences of organelles and facilitates dye and fixation free images to that are amenable to quantitation in living cells. As lipid droplets exhibit the highest refractive index of all organelles in normal, healthy cells, we used Nanolive imaging to quantify lipid content per cell. Fig. 3C shows example images of UMUC3 cells treated with DMSO or xanthohumol for 0.5–20 h post-treatment (videos available in supplemental material) and label free images to quantify lipid droplets. We observed a significant decrease in the amount of lipid droplets in xanthohumol treated cells by 20 hrs and no change in lipids following DMSO treatment. We observed a decrease in both 5637 and UMUC3 cells (Fig. 3D), although the observed effect was far greater in UMUC3, and agrees with our screening data since xanthohumol was the most toxic these cells. Fig. 3E shows Nanolive quantitation of decreased lipid content only following xanthohumol exposure. To confirm our findings, we treated UMUC3 cells with xanthohumol or deguelin for 24 h followed by lipid droplet staining with Bodipy. We observed a significant decrease in lipid droplets per cell (total lipid) following xanthohumol, but not in DMSO or deguelin treated cells (Supplemental Figure 3B). We also observed a similar loss of lipid content in 5637 cells treated with xanthohumol versus DMSO treatments (Supplemental Figure 3C). Collectively, these data agree with previous reports that showed xanthohumol treatments can suppress formation of lipid droplets, possibly through repression of LXR signaling [29] or its role as a DGAT inhibitor [30]. Our data showing marked changes in both lipid and mitochondrial features following xanthohumol treatment (Supplemental Figure 2B) suggests a key role in altering energy production.

### Trastuzumab sensitizes cells to Xanthohumol

Erb-B2 Receptor Tyrosine Kinase 2 (HER2) is a druggable target for HER2-positive breast cancer commonly treated with therapeutic antibody trastuzumab. HER2 is also expressed in a subset of bladder cancer patients and its levels are linked to prognosis [31], however HER2 inhibition with trastuzumab has been unsuccessful in treating bladder cancer in clinical trials [31,32]. Therefore, we wanted to see if any of set of toxic flavonoids had a combinatorial effect with trastuzumab by treating 5637 and UMUC3 cells with trastuzumab and a dose response of flavonoid. While trastuzumab itself did not decrease cell number, treatment with trastuzumab sensitized both 5637 and UMUC3 cells to xanthohumol (Supplemental Figure 3D and 3E, respectively), allowing xanthohumol to disrupt proliferation at a 10-fold lower concentration. Our CP analysis suggests that xanthohumol affects mitochondria and our Nanolive imaging shows a direct effect on lipid content, so the effect observed here could be the results of disrupting ATP production combined with a decrease in proliferative signals induced by ERBB2.

### Cancer cell specific toxicity of flavonoids in 3D spheroids

**Fig. 4.**
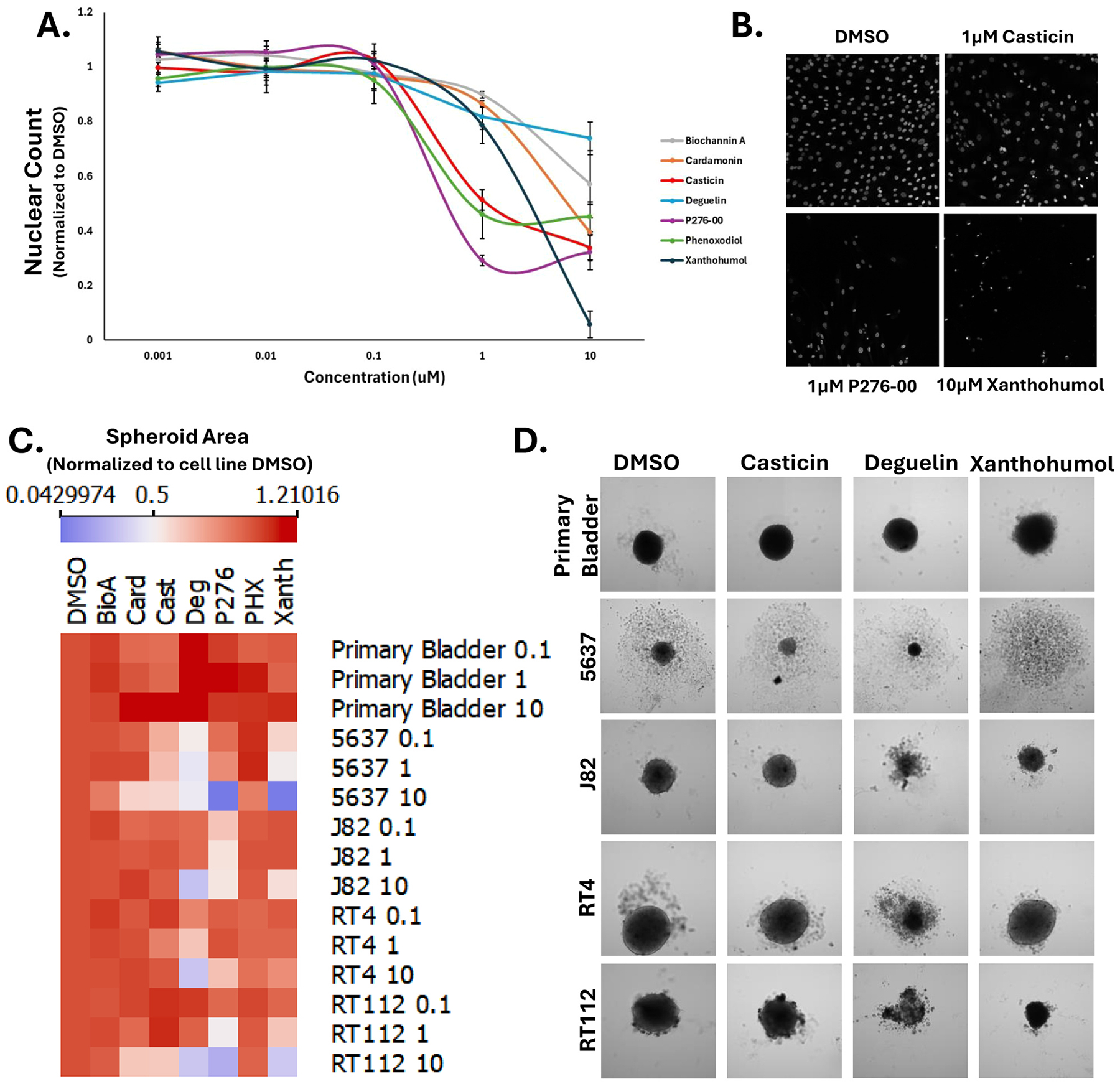
Flavonoid toxicity on bladder spheroids. A) Dose response curve of toxic flavonoids in primary bladder fibroblast cells. B) DAPI images of DMSO, casticin, P276–00, and xanthohumol. C) Heat map of spheroid area normalized to DMSO across spheroid type and compound dose. D) Example images of bladder cell lines spheroids.

Following our CP studies with 7 toxic flavonoids, we next sought to determine if they similarly affect non-cancerous bladder cells. To accomplish this, we treated a primary bladder fibroblast cell line, PCS-420–013, with our set of 7 toxic flavonoids for 5 days in a a dose response experiment (1nM-10uM). Whereas none of the flavonoids tested resulted in a decrease of cell numbers below 1uM, all caused at least a 25 % decrease in cell number at the highest dose (Fig. 4A–B). This data suggests that at least in 2D cell cultures, these flavonoids are toxic to even non-cancerous bladder cells lines.

As a follow up study, we were interested in determining how 3D cultures would respond to flavonoids shown to be toxic in 2D culture. Using a method developed by the Brenner lab [33], we first set out to see whether our bladder cancer cell lines formed spheroids. We tested whether primary fibroblasts and our original set of bladder cancer cell lines (5637, RT4, and UMUC3), and included a second set including J82 (another basal/squamous type), RT112 (another luminal P type), and UMUC13 (another neuroendocrine type) for comparison. All cells were grown in an agarose well for 5 days and treated. Supplemental Figure 3 shows that all cell lines tested formed spheroids of varying sizes except for UMUC3 and UMUC13.

Next, we examined how our set of flavonoids affected the 3D cultures. Spheroids, plated 24 hours earlier, were treated with flavonoids at 3 doses (100 nM, 1uM, 10uM) for 6 days. Spheroid area was then measured and normalized to pre-treated spheroid size. Fig. 4C shows that all flavonoids, except for phenoxodiol and biochannin A, significantly decreased the spheroid diameters at a mid-Z height confocal plane in a dose-dependent manner. Fig. 4D provides example images of the spheroids under various conditions. Interestingly, unlike the 2D culture, the primary bladder fibroblast cell line was not significantly affected by any of the flavonoids when grown as spheroids. Deguelin and P276–00 were the only compounds toxic to all cancer cell spheroids based upon reduced size and/or marked obliteration, while xanthohumol and casticin were effect against 5637, J82, and RT112, but not RT4 spheroids. Phenoxodiol and biochannin A were largely ineffective, while cardamonin was only effective at its highest dose against 5637 and RT112. These data show not only major differences in compound effectiveness in 2D vs 3D cell culture but directed us towards further testing flavonoid hits in a more complex model system.

### Xanthohumol disrupts tumors in chorioallantoic membrane models

**Fig. 5.**
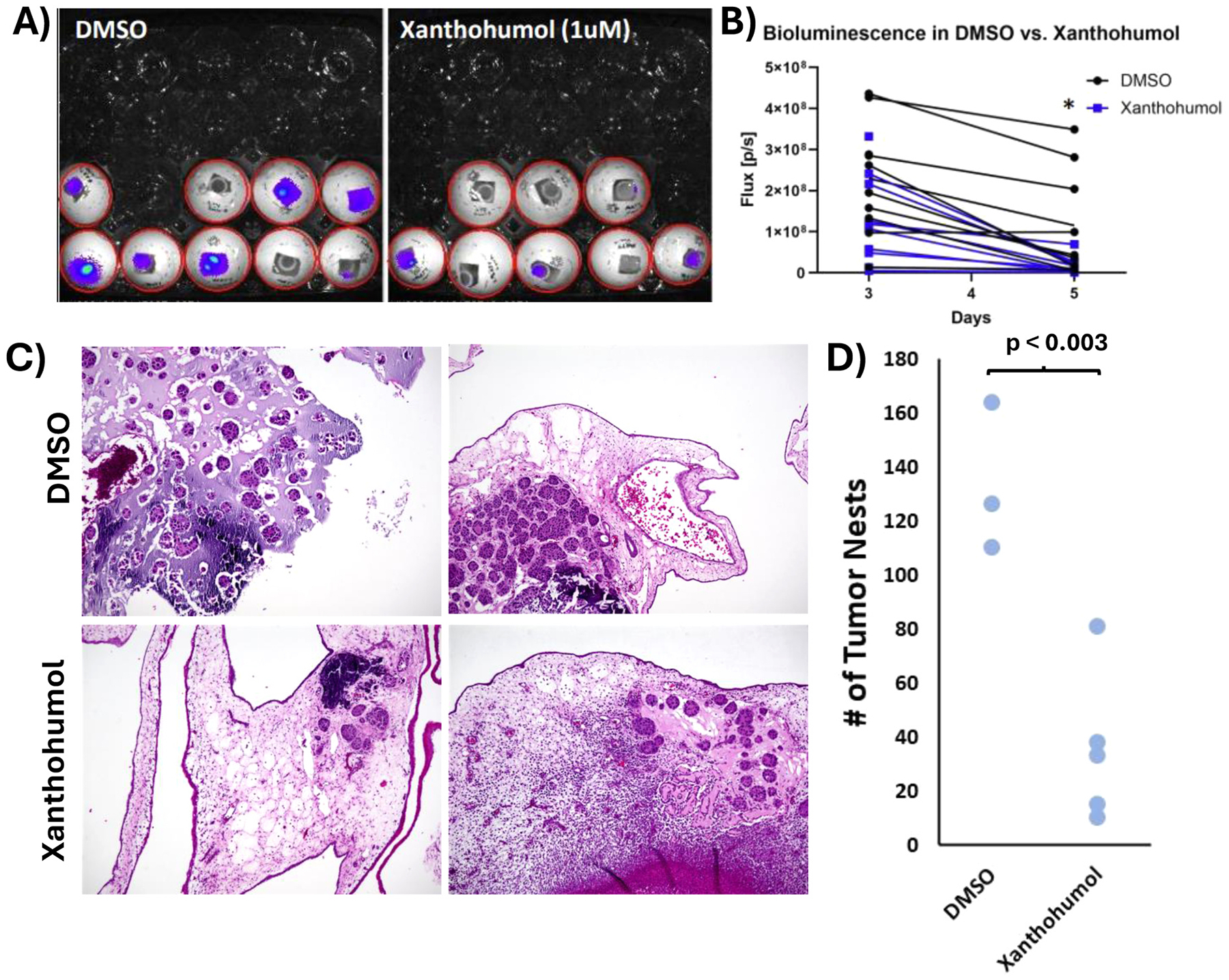
Chorioallantoic membrane models show effective Xanthohumol treatment of bladder cancer. A) IVIS images of CAM egg models with RT4 tumors treated with DMSO or xanthohumol. **B)** Quantitation of the bioluminescence of images in A. **C)** Two representative images of H&E-stained CAM tumors from DMSO or xanthohumol treated tumors. **D)** Quantitation of the number of tumor nests per egg (manual count). * represents p < 0.05.

To further our understanding of the effect of hit flavonoids in a more physiological model system we used the chorioallantoic membrane model with fertilized chicken eggs that were implanted with bladder cells to quantify tumor growth in an angiogenic environment. We tested 5637 and RT4 cells for their ability to work in this system and found that while both implanted, only the RT4 cells caused a robust tumorigenic response. We implanted RT4 cells in eggs and treated with DMSO for xanthohumol for 5 days followed by exposure with D-luciferin and imaged on an IVIS Lumina 3 in vivo imaging system. Fig. 5A shows images from the Lumina 3 system for DMSO and xanthohumol treated eggs. We observed a significant reduction in luminescence between the DMSO and xanthohumol treated samples (Fig. 5B). Following 7 days of growth, the samples were fixed, paraffin-embedded, sectioned, and H&E stained for phenotypic observation of the tumors. Fig. 5C shows representative fields from 3 different embedded tumor regions from DMSO or xanthohumol. We next manually counted the number of tumor nests in the samples and saw significantly more tumor nests in the DMSO-treated samples compared to the xanthohumol-treated eggs (Fig. 5D). These data agree with the bioluminescence data and confirms that xanthohumol can inhibit RT4 tumor growth in the CAM model. While Xanthohumol has been shown to be effective against other types of cancer [34, 35], our data shows that may be an amenable target for pharmaceutical intervention of bladder cancer.

## Discussion

The research presented here further potentiates the usefulness of Cell Painting-based phenotypic screening, and its ability to provide insights into mechanism of action and further downstream experimentation. Our findings of nuclear dysmorphia (changes to nuclear shape, texture, or brightness features) with certain flavonoids, but not others, led to the visualization of yH2AX foci accumulation following flavonoid treatment. Casticin, one of the yH2AX inducers, has previously been shown to induce DNA damage and cell viability in the TSGH-8301 bladder cancer cell line [36], further validated our findings. Casticin also has been shown to reduce expression of DNA repair machinery such as MDC1 and p-ATM [26]. Phenoxodiol is also known to induce DNA damage in metastatic prostate cells [37] and P276–00 is a CDK inhibitor known to elicit a large DNA damage response [38]. These findings further support CP screening results and can provide clues to the mechanism of action as shown previously [39].

We also observed compounds that caused toxicity but had little/no effect on DNA features including deguelin, cardamonin, biochannin A, and xanthohumol. Deguelin, a compound related to rotenone (which also showed detrimental effects on mitochondria), has been previously reported to inhibit mitochondrial complex I [40], and are used, along with rotenone, in rat models of Parkinsons disease [41]. Further, deguelin can induce apoptosis through inhibition of mitochondrial bioenergetics in cutaneous squamous cell carcinoma cell lines [42]. Both rotenone and deguelin have been tested in clinical trials, and exhibited various degrees of toxicity; however, a deguelin derivative, SH-14, has shown less toxicity with similar efficacy of binding to HSP90 [43], the purported target of deguelin and rotenone. Our 3D culture of primary bladder fibroblasts showed very little toxic effect from deguelin treatment yet was very toxic to the four bladder cancer spheroids. Combined with the ability to treat patients via a catheter versus the bloodstream, these compounds appear to be good candidates for bladder cancer intervention in animal models.

Xanthohumol caused increased mitochondrial area and intensity but not as extensive as with rotenone. This difference led us to determine how xanthohumol decreased bladder cancer cell viability, which we observed as a decrease in lipid metabolism in cancer cells and a stark decrease in the number of lipid droplets per cell. Previous reports show xanthohumol causes metabolic changes in normal physiology [44] and potentially explains its effectiveness in the loss of ATP generated from fats. Xanthohumol, found in hops that are commonly brewed into beer [35] at levels of up to 0.96 mg/L [45] are able to kill bladder cancer cells presumably through reduction in lipid, and may suggest why demonstrate it synergistically working with trastuzumab. Trastuzumab blocking Her2 activation of the PI3K-AKT-mTOR pathway combined with reduced ATP due to loss of lipids may be a combination that bladder cancer cells cannot survive. These data also suggest that a retrospective study may be illuminating to determine the occurrence of bladder cancer in patients who frequently consume hops-rich beers.

BCG therapy is an immunological intrabladder treatment instilling *Mycobacterium bovis* to activate the immune system in order to aid in the attack of tumors [46]. Combination of BCG therapy with chemotherapy is currently being studied as a treatment for NIMBC, and the addition of flavonoids described here may suggest it would also be useful in combination. In this case, the flavonoids casticin and deguelin may directly kill the cancer cells in situ whereas addition of xanthohumol could reduce tumor cell lipid stores that further weaken tumor metabolism and enhances BCG treatments to further act against the tumor. Collectively, new pheno-mechanistic studies by Cell Painting have greatly augmented the utility of large screens and hit determination, and subsequent follow-up studies that may aid the therapeutics research community in their fight against cancer and other diseases.

## Conclusion

In conclusion, our study further demonstrated the power of utilizing phenotypic screening in the search for novel therapeutics for cancer. Flavonoids are compounds that are enriched in plant life and several of the tested compounds are found in plants that we utilize for food and drink. The compounds may be naturally protective against different types of diseases and now can be utilized by phenotypic screening as we have done here. Future studies could explore retroactive datasets for a connection between drinking hoppy beverages and less bladder cancer. Taking the compounds we have discovered in this study and using them in mouse models to study their efficacy would be another excellent approach. Xenograft cancer models as well as tolerance studies in the mouse bladder could afford an answer to the legitimacy of these compounds as therapeutic agents. Xanthohumol is currently under trials for use in human subjects, but as of writing, that study is still underway, though studies have shown high levels (up to 50uM) of xanthohumol in human urine. Chicken embryos in the CAM model do have an immune system that is developing during the time of our experiment but is not fully developed until day 18, while our experiments end on day 17, so further studies of our compounds interaction with immune environments would be useful.

## Supplementary Material

1

2

## Data Availability

Data will be made available on request.

## Appendix A. Supporting information

Supplementary data associated with this article can be found in the online version at doi:10.1016/j.prenap.2025.100236.

## References

[1] Matulewicz RS, Steinberg GD, Non-muscle-invasive bladder cancer: overview and contemporary treatment landscape of neoadjuvant chemoablative therapies, Rev. Urol 22 (2020) 43–51. 32760227 PMC7393683

[2] Goldberg IP, Lichtbroun B, Singer EA, Ghodoussipour S, Pharmacologic therapies for non-muscle invasive bladder cancer: current and future treatments, Arch. Pharm. Ther 4 (2022) 13–22. PMC9431226 36051251

[3] Yang Y, Wang C, Li Z, Lu Q, Li Y, Precise diagnosis and treatment of non-muscle invasive bladder cancer – a clinical perspective, Front Oncol 13 (2023) 1042552. 36798814 10.3389/fonc.2023.1042552 PMC9927396

[4] Yanagisawa T, , Repeat transurethral resection for non-muscle-invasive bladder cancer: an updated systematic review and meta-analysis in the contemporary era, Eur. Urol. Focus 10 (2024) 41–56. 37495458 10.1016/j.euf.2023.07.002

[5] Ślusarczyk A, Zapała P, Zapała Ł, Borkowski T, Radziszewski P, Cancer-specific survival of patients with non-muscle-invasive bladder cancer: a population-based analysis, Ann. Surg. Oncol 30 (2023) 7892–7902. 37578604 10.1245/s10434-023-14051-9 PMC10562346

[6] Park JC, Citrin DE, Agarwal PK, Apolo AB, Multimodal management of muscle-invasive bladder cancer, Curr. Probl. Cancer 38 (2014) 80–108. 25087173 10.1016/j.currproblcancer.2014.06.001 PMC4190161

[7] Rinaldetti S, , High-content drug discovery targeting molecular bladder cancer subtypes, Int J. Mol. Sci 23 (2022) 10605. 36142576 10.3390/ijms231810605 PMC9506379

[8] Zhang Q, , Tectoridin inhibits the growth of bladder cancer by regulating PI3K/MAPK pathway through RAB27B, Mol. Carcinog 63 (2024) 1106–1116. 38441297 10.1002/mc.23712

[9] Su Y, Chen L, Yang J, Hesperetin inhibits bladder cancer cell proliferation and promotes apoptosis and Cycle Arrest by PI3K/AKT/FoxO3a and ER stress-mitochondria pathways, Curr. Med Chem (2024), 10.2174/0109298673283888231217174702. 38357946

[10] Arzuk E, Armağan G, Genistein and daidzein induce ferroptosis in MDA-MB-231 cells, J. Pharm. Pharm rgae106 (2024), 10.1093/jpp/rgae106. 39245043

[11] Dong X, , Baicalin induces cell death of non-small cell lung cancer cells via MCOLN3-mediated lysosomal dysfunction and autophagy blockage, Phytomedicine 133 (2024) 155872. 39096542 10.1016/j.phymed.2024.155872

[12] Kamran S, Sinniah A, Chik Z, Nelli G, Alshawsh MA, Synergistic anti-tumorigenic effect of diosmetin in combination with 5-fluorouracil on human colon cancer xenografts in nude mice, Biochem Biophys. Res Commun 735 (2024) 150677. 39265366 10.1016/j.bbrc.2024.150677

[13] Bredsdorff L, , Urinary flavonoid excretion and risk of acute coronary syndrome in a nested case-control study, Am. J. Clin. Nutr 98 (2013) 209–216. 23697704 10.3945/ajcn.112.046169

[14] Stossi F, , SPACe (Swift Phenotypic Analysis of Cells): an open-source, single cell analysis of Cell Painting data, bioRxiv (2024) 2024.03.21.586132, 10.1101/2024.03.21.586132. PMC11585637 39580445

[15] Pérez-Montero A, Zaragoza O, Luque A, Hortelano S, Acebo P, Visualization of lipid droplets in the alveolar macrophage cell line MH-S with live-cell imaging by 3D holotomographic microscopy (Nanolive), Bio Protoc 13 (2023) e4629. 10.21769/BioProtoc.4629 PMC9993077 36908642

[16] McKenna MK, , Mesenchymal stromal cell delivery of oncolytic immunotherapy improves CAR-T cell antitumor activity, Mol. Ther 29 (2021) 3529–3533. 34706248 10.1016/j.ymthe.2021.10.007 PMC8636171

[17] Li M, , The In Ovo Chick Chorioallantoic Membrane (CAM) assay as an efficient xenograft model of hepatocellular carcinoma, J. Vis. Exp (2015) 52411, 10.3791/52411. 26484588 PMC4692648

[18] Villanueva H, , Characterizing treatment resistance in muscle invasive bladder cancer using the chicken egg chorioallantoic membrane patient-derived xenograft model, Heliyon 8 (2022) e12570. 36643309 10.1016/j.heliyon.2022.e12570 PMC9834740

[19] Eray A, Erkek-Özhan S, Classification of bladder cancer cell lines according to regulon activity, Turk. J. Biol 45 (2021) 656–666. 35068946 10.3906/biy-2107-72 PMC8733949

[20] Tamashunas AC, , High-throughput gene screen reveals modulators of nuclear shape, Mol. Biol. Cell 31 (2020) 1392–1402. 32320319 10.1091/mbc.E19-09-0520 PMC7353136

[21] Zhang M, , CDK inhibitors in cancer therapy, an overview of recent development, Am. J. Cancer Res 11 (2021) 1913–1935. 34094661 PMC8167670

[22] Li X-Z, Yi L-T, Sun Q-Y, Xu C-L, Yin S, Flavopiridol inhibits oocyte maturation, reduces oocyte quality and blocks cumulus cell function, Toxicol. Lett (2024), 10.1016/j.toxlet.2024.09.002. 39276810

[23] Ibarra-Gutíerrez MT, Serrano-García N, Alcaraz-Zubeldia M, Pedraza-Chaverri J, Orozco-Ibarra M, An exploratory study on the ability of manganese to supplement rotenone neurotoxicity in rats, Brain Res 1839 (2024) 149017. 38768935 10.1016/j.brainres.2024.149017

[24] Yong SJ, Veerakumarasivam A, Teoh SL, Lim WL, Chew J, Lactoferrin Protects Against Rotenone-Induced Toxicity in Dopaminergic SH-SY5Y Cells through the Modulation of Apoptotic-Associated Pathways, J. Mol. Neurosci 74 (2024) 88. 39297981 10.1007/s12031-024-02267-7

[25] Nagelkerke A, Span PN, Staining against phospho-H2AX (γ-H2AX) as a marker for DNA damage and genomic instability in cancer tissues and cells, Adv. Exp. Med Biol 899 (2016) 1–10. 27325258 10.1007/978-3-319-26666-4_1

[26] Cheng Z-Y, , Casticin induces DNA damage and affects DNA repair associated protein expression in human lung cancer A549 cells (Running Title: Casticin Induces DNA Damage in Lung Cancer Cells), Molecules 25 (2020) 341. 31952105 10.3390/molecules25020341 PMC7024307

[27] Fu C, Zhang K, Wang M, Qiu F, Casticin and chrysosplenol D from Artemisia annua L. induce apoptosis by inhibiting topoisomerase IIα in human non-small-cell lung cancer cells, Phytomedicine 100 (2022) 154095. 35398735 10.1016/j.phymed.2022.154095

[28] Saunders N, , Dynamic label-free analysis of SARS-CoV-2 infection reveals virus-induced subcellular remodeling, Nat. Commun 15 (2024) 4996. 38862527 10.1038/s41467-024-49260-7 PMC11166935

[29] Chen S-F, Chen P-Y, Hsu H-J, Wu M-J, Yen J-H, Xanthohumol Suppresses Mylip/Idol Gene Expression and Modulates LDLR Abundance and Activity in HepG2 Cells, J. Agric. Food Chem 65 (2017) 7908–7918. 28812343 10.1021/acs.jafc.7b02282

[30] Tabata N, Ito M, Tomoda H, Omura S, Xanthohumols, diacylglycerol acyltransferase inhibitors, from Humulus lupulus, Phytochemistry 46 (1997) 683–687. 9366096 10.1016/s0031-9422(97)00157-x

[31] Zhou L, , HER2 expression associated with clinical characteristics and prognosis of urothelial carcinoma in a Chinese population, Oncologist 28 (2023) e617–e624. 36971495 10.1093/oncolo/oyad070 PMC10400138

[32] Qu M, , Advances in HER2-targeted treatment for advanced/metastatic urothelial carcinoma, Bladder (San. Fr.) 10 (2023) e21200012. 10.14440/bladder.2023.871 PMC10752798 38155921

[33] McKenna MK, , Novel banana lectin CAR-T cells to target pancreatic tumors and tumor-associated stroma, J. Immunother. Cancer 11 (2023) e005891. 36653070 10.1136/jitc-2022-005891 PMC9853244

[34] Pérez-Valero Á, , Antitumor effect and gut microbiota modulation by quercetin, luteolin, and xanthohumol in a rat model for colorectal cancer prevention, Nutrients 16 (2024) 1161. 38674851 10.3390/nu16081161 PMC11054239

[35] Tejero A, León-Navarro DA, Martín M, Effect of xanthohumol, a bioactive natural compound from hops, on adenosine pathway in rat C6 glioma and human SH-SY5Y neuroblastoma cell lines, Nutrients 16 (2024) 1792. 38892725 10.3390/nu16111792 PMC11174739

[36] Huang A-C, , Casticin induces DNA damage and impairs DNA repair in human bladder cancer TSGH-8301 Cells, Anticancer Res 39 (2019) 1839–1847. 30952724 10.21873/anticanres.13291

[37] Aguero MF, Venero M, Brown DM, Smulson ME, Espinoza LA, Phenoxodiol inhibits growth of metastatic prostate cancer cells, Prostate 70 (2010) 1211–1221. 20564423 10.1002/pros.21156

[38] Maude SL, Enders GH, Cdk inhibition in human cells compromises chk1 function and activates a DNA damage response, Cancer Res 65 (2005) 780–786. 15705874

[39] Chandrasekaran SN, , Three million images and morphological profiles of cells treated with matched chemical and genetic perturbations, Nat. Methods 21 (2024) 1114–1121. 38594452 10.1038/s41592-024-02241-6 PMC11166567

[40] Naguib A, , Mitochondrial complex i inhibitors expose a vulnerability for selective killing of Pten-null cells, Cell Rep 23 (2018) 58–67. 29617673 10.1016/j.celrep.2018.03.032 PMC6003704

[41] Caboni P, , Rotenone, deguelin, their metabolites, and the rat model of Parkinson’s disease, Chem. Res Toxicol 17 (2004) 1540–1548. 15540952 10.1021/tx049867r

[42] Hail N, Lotan R, Apoptosis induction by the natural product cancer chemopreventive agent deguelin is mediated through the inhibition of mitochondrial bioenergetics, Apoptosis 9 (2004) 437–447. 15192326 10.1023/B:APPT.0000031449.57551.e1

[43] Kim W-Y, , A novel derivative of the natural agent deguelin for cancer chemoprevention and therapy, Cancer Prev. Res (Philos.) 1 (2008) 577–587. 10.1158/1940-6207.CAPR-08-0184 PMC2738643 19139008

[44] Miranda CL, , Xanthohumol improves dysfunctional glucose and lipid metabolism in diet-induced obese C57BL/6J mice, Arch. Biochem Biophys 599 (2016) 22–30. 26976708 10.1016/j.abb.2016.03.008 PMC4875845

[45] Chen Q-H, , Preparative isolation and purification of xanthohumol from hops (Humulus lupulus L.) by high-speed counter-current chromatography, Food Chem 132 (2012) 619–623. 26434340 10.1016/j.foodchem.2011.10.098

[46] Serretta V, BCG and bladder cancer. Forty-eight years after Morales report, Urologia 91 (2024) 459–467. 38757638 10.1177/03915603241252909

