# Supplementary material for: A phenotypic screen identifies xanthohumol and other flavonoids as killers of bladder cancer
Source: Pharmacol Res Nat Prod. Author manuscript; Available in PMC 2026 May 29. (PMC13218733; doi:10.1016/j.prenap.2025.100236)

Figure S1. Actinomycin D and Berberine chloride as screen controls.

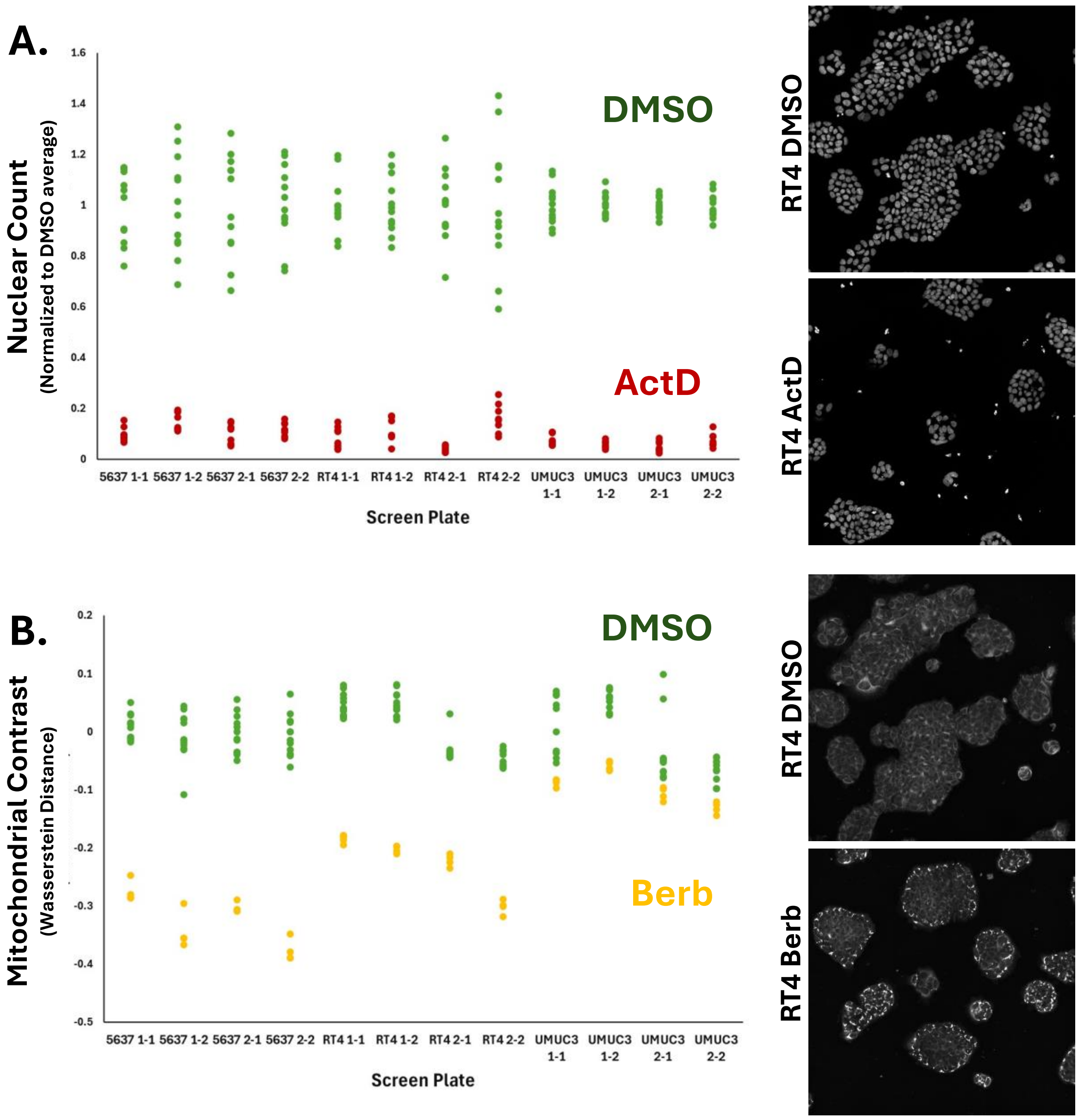

Figure S2. Cell painting features give insight into mechanism of toxicity

A.

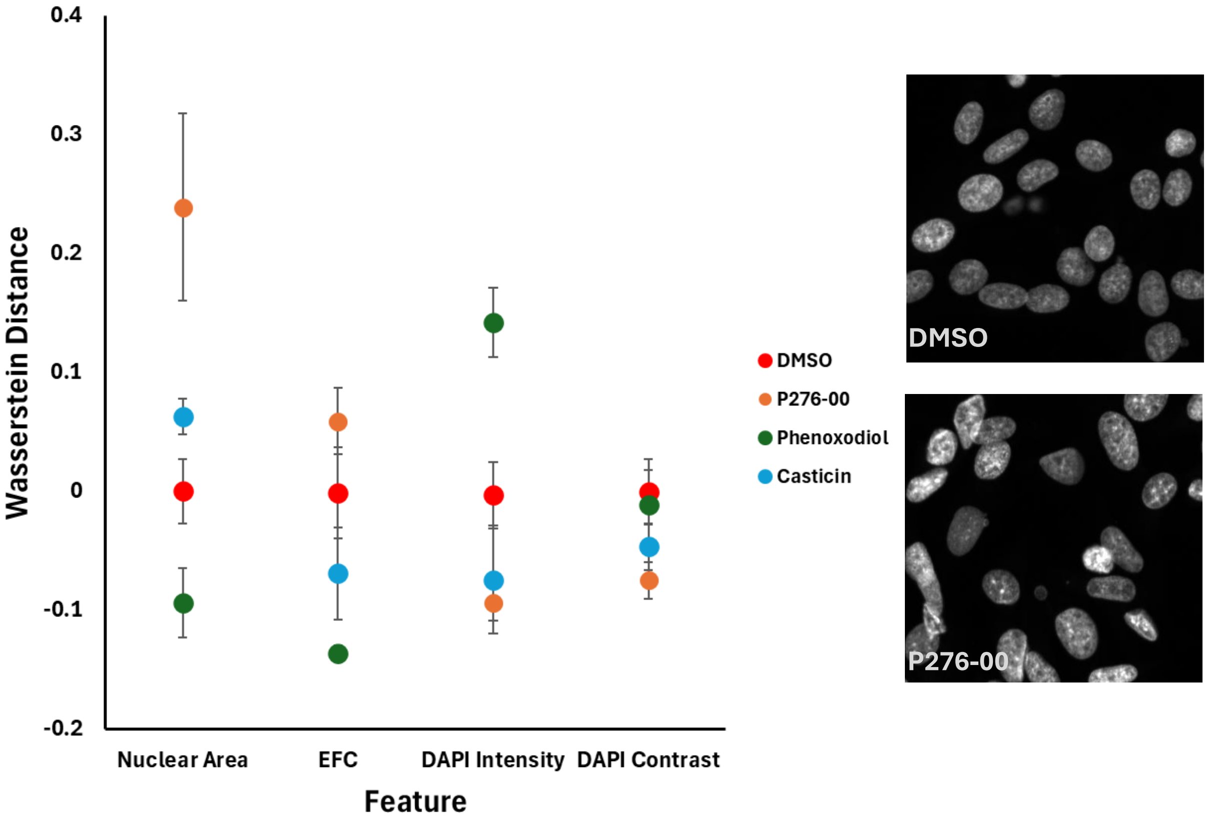

B.

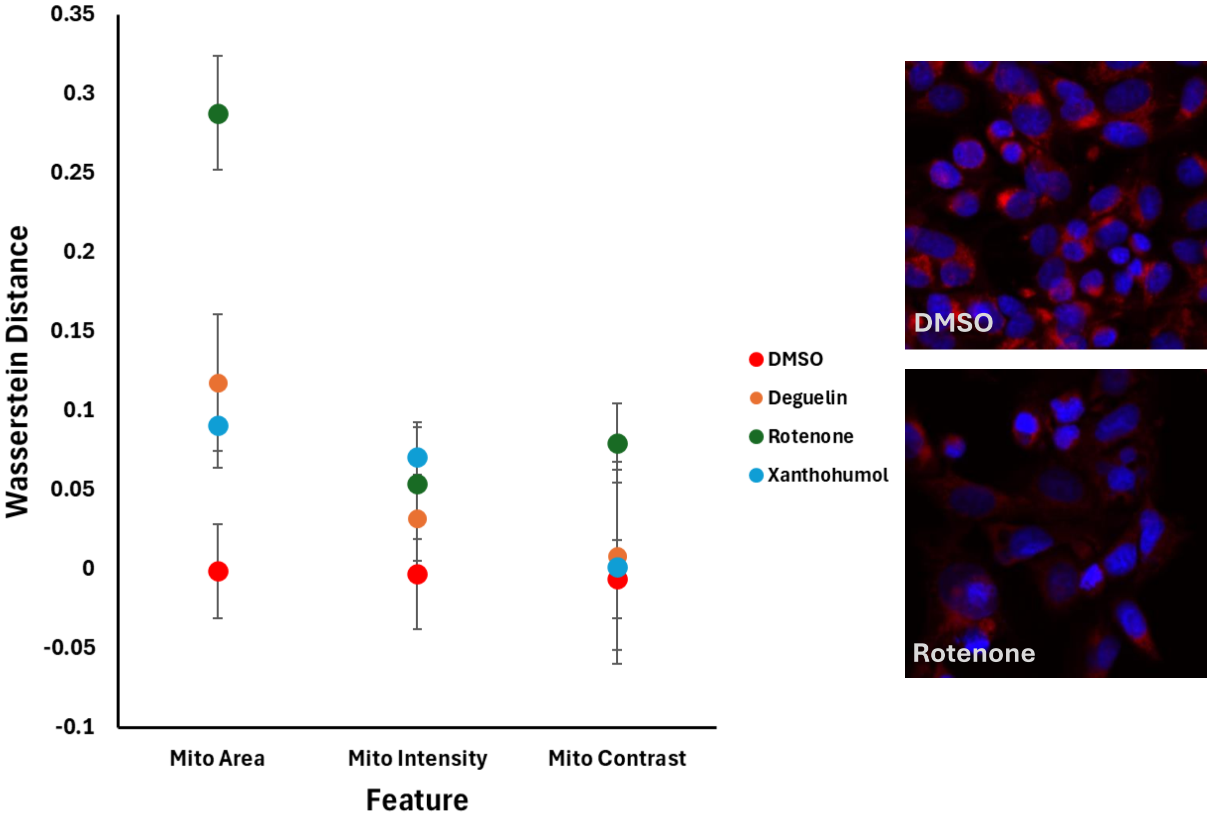

Figure S3.

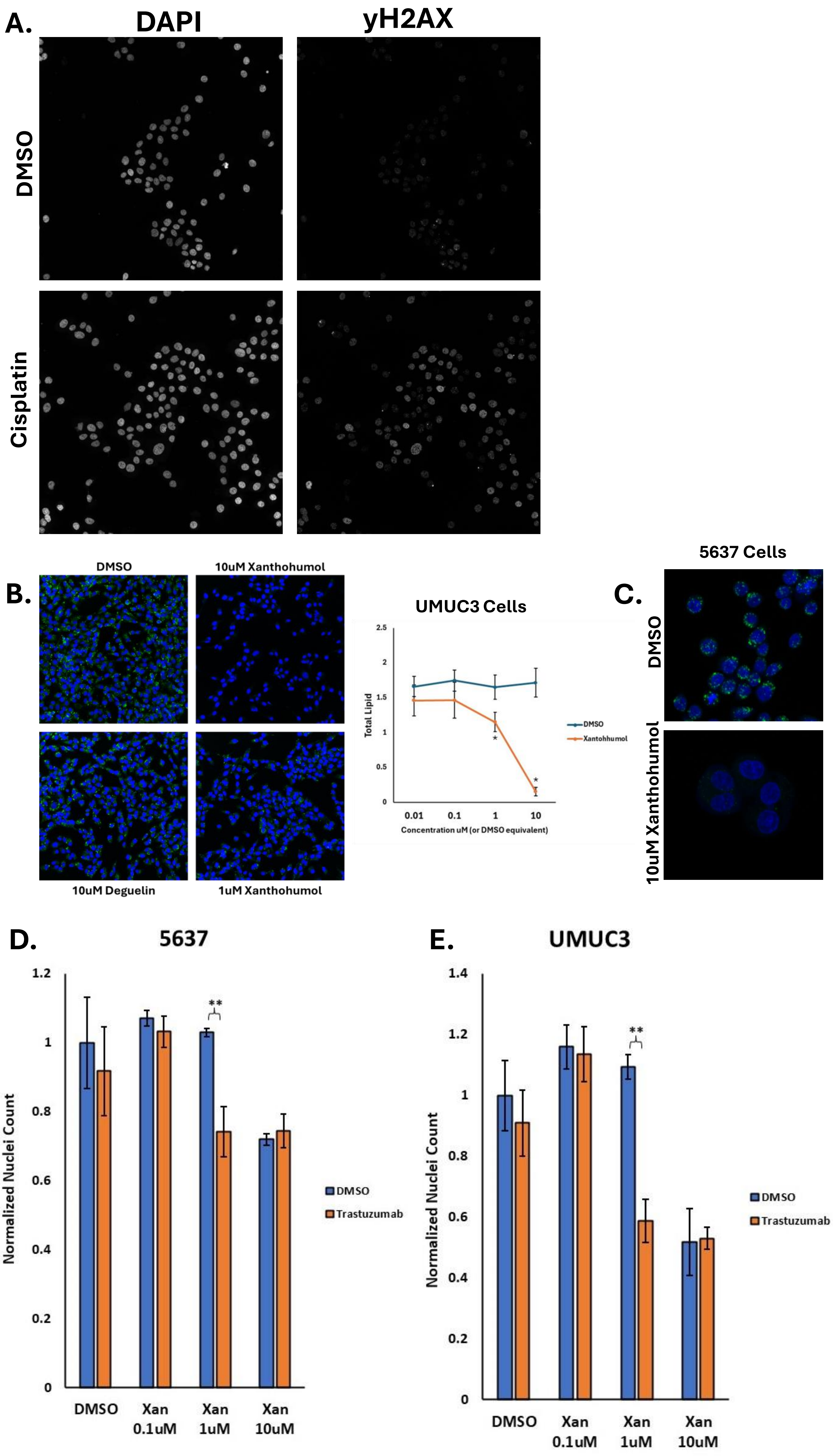
A.
DAPI
yH2AX
DMSO
Cisplatin
B.
DMSO
10uM Xanthohumol
10uM Deguelin
1uM Xanthohumol
UMUC3 Cells
Total Lipid
Concentration uM (or DMSO equivalent)
DMSO
Xantohhumol
C.
5637 Cells
DMSO
10uM Xanthohumol
D.
5637
Normalized Nuclei Count
DMSO
Xan 0.1uM
Xan 1uM
Xan 10uM
DMSO
Trastuzumab
E.
UMUC3
Normalized Nuclei Count
DMSO
Xan 0.1uM
Xan 1uM
Xan 10uM
DMSO
Trastuzumab

Figure S4. Bladder Sphere Images

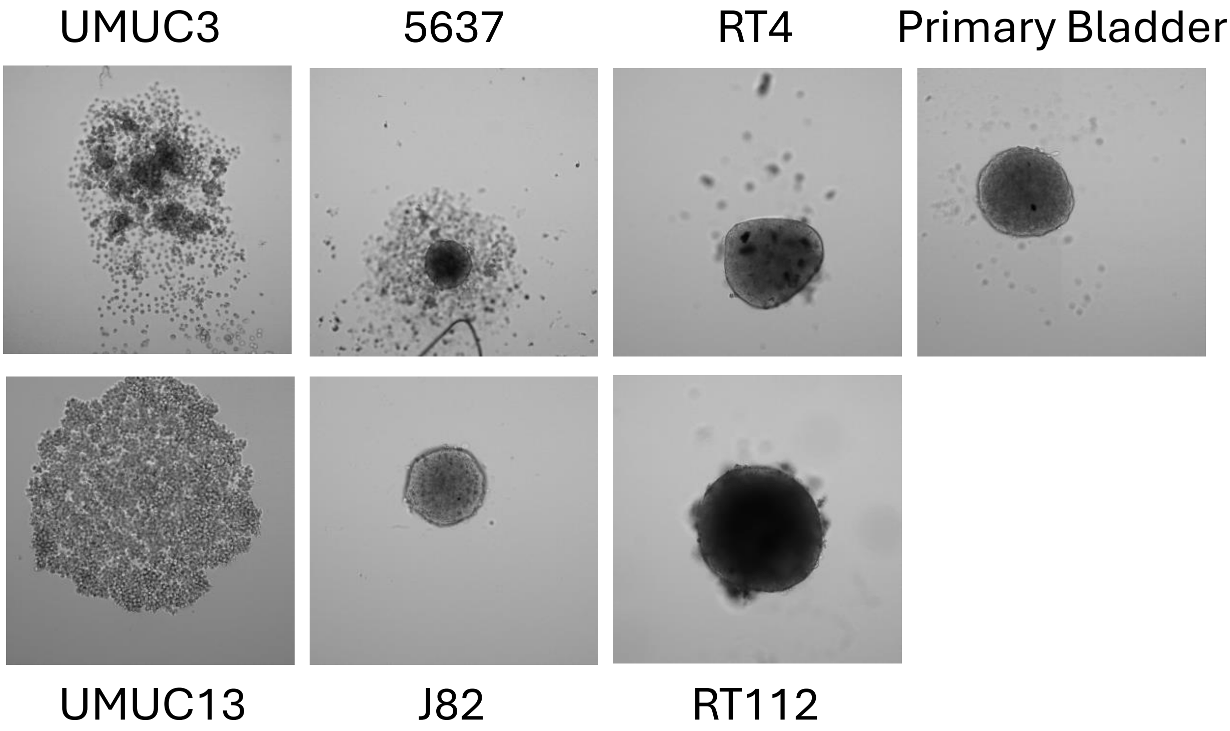

Supplement: 1 [file NIHMS2175013-supplement-1.pdf]
