# Supplementary material for: A phenotypic screen identifies xanthohumol and other flavonoids as killers of bladder cancer
Source: Pharmacol Res Nat Prod. Author manuscript; Available in PMC 2026 May 29. (PMC13218733; doi:10.1016/j.prenap.2025.100236)
Supplement: 2 [file NIHMS2175013-supplement-2.docx]

**Supplemental Figure 1. Actinomycin D and berberine chloride as screening controls.** Dot plots and example images of screening controls. **A)** Dot plot of normalized nuclear count showing the replicates of DMSO (green) and actinomycin D (red) across screening plates. Images are of DMSO and actinomycin D treated RT4 cell nuclei. **B)** Dot plot of mitochondrial contrast showing the replicates of DMSO (green) and berberine chloride (yellow) across screening plates. Images are of DMSO and berberine chloride treated RT4 cell mitochondria.

**Supplemental Figure 2. Cell painting features give insight into the mechanism of toxicity**. **A)** Dot plots of flavonoid compounds and how they affect features of DAPI staining including nuclear area, EFC ratio, DAPI intensity, and DAPI contrast. **B)** Dot plots of flavonoid compounds and how they affect features of Mitotracker staining including mitochondrial area, mitochondrial intensity, and mitochondrial contrast.

**Supplemental Figure 3. Expansion of Figure 3. A)** Representative images of DMSO or cisplatin-treated UMUC3 cells. **B)** Representative images of UMUC3 cells treated with DMSO, xanthohumol or deguelin and stained with DAPI and Bodipy. Line graph depicting a decrease in the amount of lipid with UMUC3 cells with increasing doses of xanthohumol. **C)** Images of 5637 cells treated with DMSO or xanthohumol and stained with DAPI and Bodipy. **D)** Dose response of xanthohumol in 5637 cells treated with or without trastuzumab (25ug/ml). **E)** Dose response of xanthohumol in UMUC3 cells treated with or without trastuzumab (35ug/ml). * represents a p-value of less than 0.05. ** represents a p-value of less than 0.01.

**Supplemental Figure 4. Example images of bladder spheroids.** Examples images of untreated spheroids from 7 bladder cell lines.
